# Revisiting Primary Particles in Layered Lithium Transition‐Metal Oxides and Their Impact on Structural Degradation

**DOI:** 10.1002/advs.201800843

**Published:** 2019-01-25

**Authors:** Seung‐Yong Lee, Gyeong‐Su Park, Changhoon Jung, Dong‐Su Ko, Seong‐Yong Park, Hee Goo Kim, Seong‐Hyeon Hong, Yimei Zhu, Miyoung Kim

**Affiliations:** ^1^ Department of Materials Science and Engineering and Research Institute of Advanced Materials Seoul National University Seoul 08826 Republic of Korea; ^2^ Condensed Matter Physics and Materials Science Department Brookhaven National Laboratory Upton NY 11973 USA; ^3^ AE Group Platform Technology Lab Samsung Advanced Institute of Technology Suwon‐si Gyeonggi‐do 443‐803 Republic of Korea

**Keywords:** layered lithium transition‐metal oxide, lithium‐ion batteries, mechanical cracks, primary particles, transmission electron microscopy

## Abstract

Layered lithium transition‐metal oxide materials, e.g., Li(Ni_1−_ *_x_*_−_ *_y_*Co*_x_*Mn*_y_*)O_2_ (NCM) and Li(Ni_1−_ *_x_*_−_ *_y_*Co*_x_*Al*_y_*)O_2_, are the most promising candidates for lithium‐ion battery cathodes. They generally consist of ≈10 µm spherical particles densely packed with smaller particles (0.1–1 µm), called secondary and primary particles, respectively. The micrometer‐ to nanometer‐sized particles are critical to the battery performance because they affect the reaction capability of the cathode. Herein, the crystal structure of the primary particles of NCM materials is revisited. Elaborate transmission electron microscopy investigations reveal that the so‐called primary particles, often considered as single crystals, are in fact polycrystalline secondary particles. They contain low‐angle and exceptionally stable special grain boundaries (GBs) presumably created during aggregation via an oriented attachment mechanism. Therefore, this so‐called primary particle is renamed as primary‐like particle. More importantly, the low‐angle GBs between the smaller true primary particles cause the development of nanocracks within the primary‐like particles of Ni‐rich NCM cathodes after repetitive electrochemical cycles. In addition to rectifying a prevalent misconception about primary particles, this study provides a previously unknown but important origin of structural degradation in Ni‐rich layered cathodes.

The demand for lithium‐ion batteries (LIBs) with higher energy densities is expected to continue increasing to keep pace with advances in electronic technology. Various layered lithium transition‐metal oxide materials (LiMO_2_; M = Ni, Co, Mn, etc.) have been suggested as viable alternatives to replace the conventional lithium cobalt oxide (LiCoO_2_) in next‐generation high‐capacity LIB cathodes, which is considered a bottleneck in the LIB development.^1^, ^2^, ^3^ In particular, Ni‐rich layered cathodes, such as Li(Ni_1−_ *_x_*_−_ *_y_*Co*_x_*Mn*_y_*)O_2_ (NCM) and Li(Ni_1−_ *_x_*_−_ *_y_*Co*_x_*Al*_y_*)O_2_ (NCA), where *x* + *y* < 0.2, have received considerable attention due to their greater specific capacity induced by the higher concentration of Ni, which is a major redox participant. However, the rapid performance degradation of Ni‐rich layered cathodes, mostly ascribed to the inherent phase instability and structural deterioration caused by surface reconstruction and large volume changes during repeated cycles, hinders their commercialization.^4^, ^5^, ^6^, ^7^, ^8^

In general, layered lithium transition‐metal oxides, including NCM and NCA, consist of 10 µm spherical particles densely packed with smaller particles (0.1–1 µm), so‐called secondary and primary particles, respectively.^4^, ^9^, ^10^ This microstructure helps the facile insertion/extraction of lithium ions into/from cathode particles.^11^ In addition, severe structural degradation, e.g., surface reconstruction and microcracks, originates from the “primary particles” during the cycles.^8^, ^12^ Accordingly, understanding the behavior of the primary particles in the layered lithium transition‐metal oxides is crucial to improve the performance of batteries. The term “primary particle” is very commonly but ambiguously used, specifically since the emergence of nonclassical nucleation and growth mechanisms such as oriented attachment, which is a well‐known crystal growth mechanism based on the alignment and aggregation of nanoparticles.^13^, ^14^, ^15^ In 2001, the National Institute of Standards and Technology proposed the definition of a primary particle as “the smallest identifiable subdivision in a particulate system.”^16^ In terms of both classical and nonclassical crystallization, primary particles are described as particles only grown from the critical crystal nucleus.^17^, ^18^, ^19^ Although it is not precisely used in related fields, the term “primary particle” is generally used to indicate the smallest unit of particles and the basis for the growth of larger particles before aggregation or agglomeration. Therefore, primary particles are often considered as single crystals.

So‐called primary particles of a few hundred nanometers in layered lithium transition‐metal oxides have also been frequently regarded as single crystals on the basis of selected area electron diffraction (SAED) and other conventional analyses.^7^, ^20^, ^21^, ^22^, ^23^ Herein, we found that most of the so‐called primary particles are in fact polycrystalline secondary particles composed of smaller well‐aligned true primary particles with low‐angle and coherent symmetrical special boundaries, as revealed by comprehensive and precise transmission electron microscopy (TEM) investigations of NCM materials. This result was valid for all examined materials, including Ni‐rich NCM. In addition to rectifying a common misconception about primary particles, our study provides strong evidence of the oriented attachment mechanism for crystal growth during the synthesis of layered lithium transition‐metal oxides.^13^, ^18^ Furthermore, we revealed that the low‐angle boundaries between the true primary particles in a primary‐like particle stimulate the development of nanocracks, which are distinguished from the microcracks developed between primary‐like particles in Ni‐rich NCM cathodes after prolonged electrochemical cycles. This indicates that a hidden but important factor aggravates the structural degradation of Ni‐rich layered cathodes and therefore draws attention to the significance of redefining the primary particles. We provide clear structural information on primary particles in layered lithium transition‐metal oxides and their impact on structural deterioration in LIBs, which also points out the best direction for the further development of Ni‐rich layered cathode materials.

We investigated NCM cathode materials with two concentration ratios of transition‐metal ions, Li(Ni_0.33_Co_0.33_Mn_0.33_)O_2_ (NCM 111) and Li(Ni_0.8_Co_0.1_Mn_0.1_)O_2_ (NCM 811), from the microscale (spherical particles) to the atomic scale (so‐called primary particles). The NCM materials were prepared by a sequence of coprecipitation and solid‐state sintering methods, which is one of the most common synthesis procedures for layered lithium transition‐metal oxide cathodes such as NCM and NCA.^24^, ^25^, ^26^ The coprecipitation method was employed to synthesize spherical particles of (Ni_1−2_ *_x_*Co*_x_*Mn*_x_*)(OH)_2_ precursors, and the subsequent solid‐state sintering of (Ni_1−2_ *_x_*Co*_x_*Mn*_x_*)(OH)_2_ and LiOH·H_2_O powders was performed to obtain the Li(Ni_1−2_ *_x_*Co*_x_*Mn*_x_*)O_2_ materials. The structural terms of layered lithium transition‐metal oxide materials, which will be thoroughly discussed in this paper, are schematically described in **Figure** **1**a. The morphologies of NCM 111 and NCM 811 were compared by scanning electron microscopy (SEM). As shown in Figure 1b–e, both NCM materials are ≈10 µm spherical particles composed of smaller, densely packed particles, the so‐called primary particles. The SEM images show that particles of the Ni‐rich NCM material (NCM 811) are slightly smaller. The atomic‐resolution high‐angle annular dark‐field (HAADF) and annular bright‐field (ABF) scanning transmission electron microscopy (STEM) images (Figure 1f) confirm the atomic structure of the NCM materials as that of an α‐NaFeO_2_‐type layered structure (*R*−3*m* space group). The atomic model structure of the NCM material along the [100] projected direction is shown in Figure 1g and is also superimposed onto the STEM images in Figure 1f. We additionally confirmed that NCM 111 and NCM 811 have the same atomic structure.

**Figure 1:**
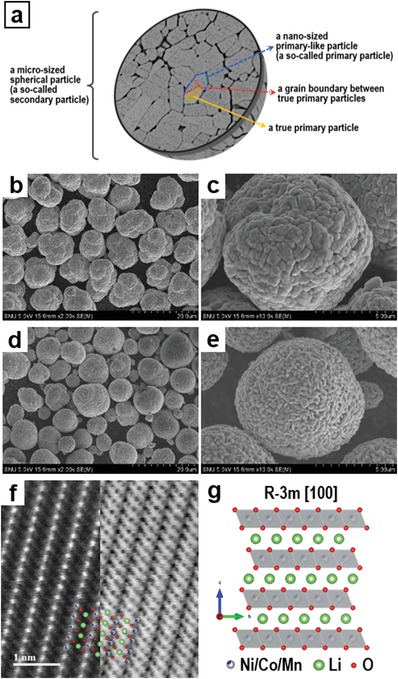
a) Description of the structural terms of layered lithium transition‐metal oxide cathode materials. SEM images of pristine b,c) NCM 111 and d,e) NCM 811 cathode materials. f) Atomic‐resolution HAADF (left) and ABF‐STEM (right) images of pristine NCM materials aligned along the [100] direction. An atomic model structure is superimposed on the image. g) Enlarged atomic model structure of the NCM material viewed along the [100] direction based on ICDD No. 00‐056‐0147.

We further investigated the so‐called primary particles of the NCM materials by elaborate electron diffraction and STEM studies to obtain a detailed structure. **Figure** **2** shows the crystallographic orientation maps and relevant analytical data of the so‐called primary particles in NCM 111 and NCM 811, which were obtained with an ASTAR tool (NanoMEGAS).^27^ The crystal orientation maps were generated and the disorientation angles inside the so‐called primary particles were measured based on precession nanobeam electron diffraction (NBD) patterns. The NBD patterns were scanned and collected for the area of interest on the sample in several nanometer steps with a TEM instrument coupled with the ASTAR tool. Figure 2a,e shows the virtual bright field (VBF) images of the cross sections of the ≈10 µm spherical particles in NCM 111 and NCM 811 obtained from the transmitted beam intensities of the scanned precession NBD patterns. Figure 2b–d,f–h shows the corresponding color‐coded crystal orientation maps that were constructed from the same scanned precession NBD patterns. The cross‐sectional TEM samples were prepared by the focused‐ion beam (FIB) technique from a spherical particle of each NCM material. In Figure 2b–d,f–h, each original crystal orientation map (Figure S1a–c,e–g, Supporting Information) was merged with the corresponding index map (Figure S1d,h, Supporting Information), which is a map of the matching index between the recorded NBD pattern and the calculated best‐fit electron diffraction pattern from the crystal structural model, by the multiplication method. The boundaries of the so‐called primary particles are better distinguished in the merged maps. The color codes for the crystal orientation maps are shown in Figure 2k. The orientation maps clearly show the large difference in the size of so‐called primary particles between the two NCM materials, which may be attributed to the relative instability of the Ni‐rich NCM material.^4^, ^5^, ^6^, ^22^

**Figure 2:**
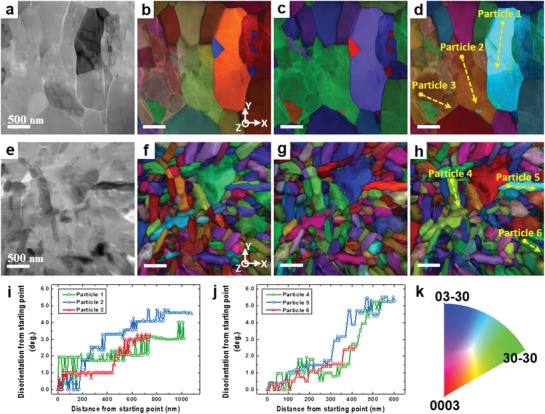
Crystallographic orientation maps and disorientation results of pristine NCM 111 and NCM 811 obtained from scanned precession NBD patterns using an ASTAR device (NanoMEGAS). VBF images from cross‐sectional TEM samples of a ≈10 µm spherical particle of a) NCM 111 and e) NCM 811. The VBF images were obtained from the transmitted beam intensities of the scanned precession NBD patterns at each scanning point. Color‐coded crystallographic orientation maps obtained from b–d) NCM 111 and f–h) NCM 811, indicating the orientation of planes normal to the b,f) *x*‐, c,g) *y*‐, and d,h) *z*‐axis. The orientation maps were constructed from the same scanned precession NBD patterns used for the VBF images shown in parts (a) and (e). Raw crystal orientation maps (Figure S1a–c,e–g, Supporting Information) were combined with index maps (Figure S1d,h, Supporting Information) to better distinguish small particles. Disorientation relationship of the grains inside the so‐called primary particles of i) NCM 111 and j) NCM 811, calculated from the scanned precession NBD data. The graphs describe the variation of the disorientation angles in each of the so‐called primary particles along the scanning lines indicated as yellow dashed arrows in parts (d) and (h), i.e., particles 1–6. Dots at the end of the arrows indicate the starting point of each scanning line and the reference orientation, i.e., 0°. k) Color codes for the crystal orientation maps expressed in the standard stereographic triangle notation.

Each so‐called primary particle shows an almost uniform grayscale contrast except for the dislocations in the VBF images (Figure 2a,e) and a single color in the crystal orientation maps (Figure 2b–d,f–h) and is therefore assumed to be a single crystal. However, interestingly, the precise disorientation analysis based on the scanned precession NBD patterns revealed several degrees of misorientation within the so‐called primary particles in both NCM materials, as shown in Figure 2i,j. The graphs show several examples of disorientation angles within each of the so‐called primary particles, which were measured along the yellow dashed arrows and marked as particles 1–6 in Figure 2d,h. The dot at the end of each arrow indicates the starting point of the disorientation scanning lines, which is set as the reference orientation, i.e., 0°. The disorientation analysis from the NBD patterns might include a ≈1° error because of the angular resolution limit of the orientation indexing in ASTAR; however, the slight misorientations in the small particles remain obvious in both NCM materials. We extracted precession NBD patterns from the starting and the end points of the disorientation scanning lines for each particle in Figure S2 in the Supporting Information, which helps to recognize the misorientations (see Supporting Information for details). Therefore, the so‐called primary particles of a few hundred nanometers, which have often been considered as single crystals,^7^, ^20^, ^21^, ^22^, ^23^ are in fact polycrystals with low‐angle grain boundaries (GBs).

We further examined the polycrystalline primary‐like particles (the so‐called primary particles) by STEM, as shown in **Figure** **3**. The particle in Figure 3a corresponds to the bright sky‐blue one marked as particle 1 in Figure 2d. The dark lines show that a single primary‐like particle contains several GBs with two proximal grains. As shown in Figure 3b, the two proximal grains of region (i) in Figure 3a have almost the same orientation, but the peak splitting of the (012) and (006) reflections in the fast Fourier‐transformed (FFT) pattern revealed a ≈2° in‐plane rotation between the grains (see Figure S3 in the Supporting Information for comparison with high‐resolution TEM (HRTEM)‐FFT patterns). The atomic‐resolution ABF‐STEM image from region (i) is shown in the inset of Figure 3b. Figure 3c,d shows other low‐angle GBs in the same primary‐like particle. The atomic‐resolution HAADF‐STEM images (Figure 3c,d) clearly show a misorientation of a few degrees between the proximal grains. In particular, the two overlapped grains with marginal in‐plane rotation along the same zone axis (Figure 3c) show moiré fringes in the low‐magnification ABF‐STEM image, as indicated by the yellow arrows in Figure 3a. The measured spacing between two dark lines of the moiré fringes of ≈25 nm agrees with the theoretical spacing that can be derived from the equation below

(1) $$D\;=\;\frac{p/2}{\sin\left(\alpha /2\right)}$$

where *D*, *p*, and α indicate the spacing between two dark (or pale) lines, the step of the patterns (*p* = *d*_{003}_ ≈ 0.45 nm), and the angle of rotation between the second pattern and the first one (α ≈ 1°), respectively.^28^ In addition to the low‐angle GBs, special boundaries were observed near the triple point, as shown in Figure 3e. Figure 3f shows that a 2°‐rotated low‐angle GB was formed between grains I and III, as also seen in Figure 3b–d. However, interestingly, a grain with a different in‐plane orientation was also observed near this triple point (denoted as II in Figure 3e). In this figure, grain II has the same zone axis family, 〈100〉, as the other grains in the primary‐like particle but a 180°‐flipped orientation [−100] and an in‐plane rotation of about 70° (Figure 3e,g). Periodic overlaps of transition‐metal ions at GBs of grains II/I and II/III created two types of coherent symmetrical special GBs of the spinel‐like structure (MgAl_2_O_4_ structure),^29^ as clearly shown in the atomic‐resolution HAADF‐STEM image in Figure 3e. These coherent symmetrical boundaries are exceptionally stable compared to other high‐angle grain boundaries because of the small lattice mismatch. The coherent symmetrical boundaries also observed in another primary‐like particle (Figure S4, Supporting Information) imply their high probability and good stability (see Supporting Information for details). Remarkably, a slight change in the interplanar spacing was also observed between these grains at the GB. Due to the crystallographic relationship, the proximal grains share several diffraction spots at the same position in the reciprocal space, e.g., the diffraction spots from the (012) plane in II and the (00−6) plane in I + III and the spots from the (006) plane in II and the (0−1−2) plane in I + III, as marked in Figure 3f,g, respectively. For precise and periodic overlaps of transition‐metal ions between the two grains at the atomic scale, the shared diffraction spots have the same interplanar spacing, resulting in tolerance to slight variations in the transition‐metal oxide (MO_2_) slab spacing. The interplanar spacing or half‐spacing of the slabs of the (006) planes is 0.22 nm in grain II and 0.23 nm in grains I and III, which represents a ≈5% difference. It might include a few percent of errors due to the low spatial resolution of the FFT patterns. However, this result still implies that under stress, the interplanar or atomic spacing in the NCM cathode material is flexible to some extent. Furthermore, the prevalence of the low‐angle and special GBs in the primary‐like particles (the so‐called primary particles) strongly suggests that oriented attachment, a well‐known nonclassical crystal growth mechanism based on the assembly and merging of primary particles with crystal lattice alignment,^13^, ^14^, ^15^ occurred during the synthesis of the NCM materials to minimize the total free energy. Without the oriented attachment, it is highly improbable that small grains become almost iso‐oriented by the mere collision during their crystal growth, forming the larger primary‐like particles including lots of tightly connected low‐angle and coherent symmetrical special GBs. The grains were not perfectly iso‐oriented during the oriented attachment, but it rather strongly supports that the polycrystalline primary‐like particles were well‐aligned aggregates. This new description of the so‐called primary particle contrasts with the typical definition of primary particles as the subunits of aggregates or as the smallest unit for growth to larger particles.^16^ Instead, each small grain (grains I, II, and III in Figure 3e) in the primary‐like particles should be regarded as a true primary particle. However, it is still possible that some of the grains are already the resultant aggregates formed by the perfect oriented attachment of much smaller grains. In addition, the size of each true primary particle in the NCM materials is similar to or larger than the general size of the so‐called primary particles of the (Ni_1−_ *_x_*_−_ *_y_*Co*_x_*Mn*_y_*)(OH)_2_ precursors.^30^, ^31^, ^32^, ^33^, ^34^ Therefore, the imperfect oriented attachment, which causes the formation of low‐angle and special GBs in the primary‐like particles of NCM materials, may have mostly occurred during the solid‐state sintering of the (Ni_1−_ *_x_*_−_ *_y_*Co*_x_*Mn*_y_*)(OH)_2_ and LiOH·H_2_O powders.

**Figure 3:**
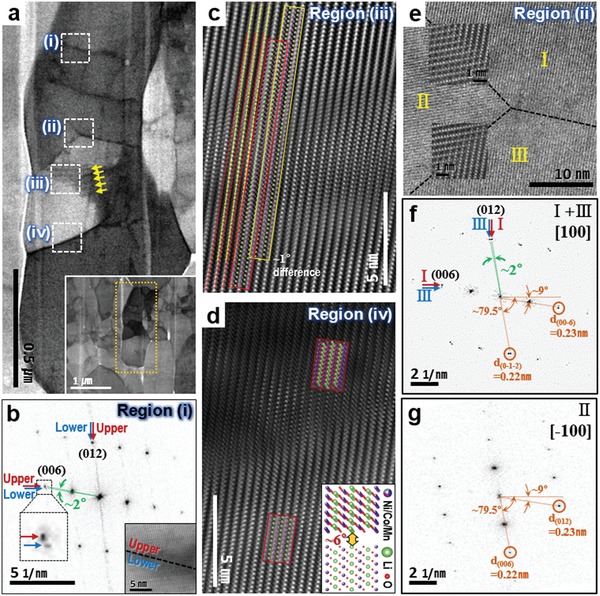
a) A representative ABF‐STEM image of a primary‐like NCM 111 particle, corresponding to the bright sky‐blue one in Figure 2d. A low‐magnification ABF‐STEM image is shown in the inset. Yellow arrows indicate the dark lines of moiré fringes. b) FFT pattern from an atomic‐resolution ABF‐STEM image (inset) of region (i). c,d) Atomic‐resolution HAADF‐STEM images of regions (iii) and (iv), respectively, with superimposed atomic model structures showing the low‐angle GBs. e) Atomic‐resolution HAADF‐STEM image near a GB triple point. Magnified HAADF‐STEM images are superimposed on the figure. Special GBs, coherent symmetrical boundaries having a MgAl_2_O_4_‐type spinel structure, are observed between grains II/I and II/III. FFT patterns of f) the GB between grains I and III, and g) grain II.

Further investigations of the mechanical degradation of the NCM materials revealed the importance of redefining the primary particles in layered lithium transition‐metal oxide cathodes. We directly compared the structural changes of both NCM 111 and 811 cathodes that occur during electrochemical cycling (from micrometer‐sized spherical particles to true primary particles) in order to study the severe structural deterioration of the Ni‐rich layered cathodes. In addition to the well‐known formation of microcracks between the primary‐like particles,^8^ we observed nanocracks within the primary‐like particles after repetitive electrochemical cycles, which can also be an important factor affecting the performance degradation of Ni‐rich layered cathodes.

**Figure** **4**a–h shows the microcrack evolution between the primary‐like particles inside the micrometer‐sized spherical particles investigated by ex situ SEM analysis. The formation of microcracks is generally accepted as the main origin of mechanical and structural degradation in Ni‐rich layered cathodes.^4^, ^5^ The spherical particles in the slurry electrode of each NCM material were cut using the FIB technique, and the material was then electrochemically cycled in a conventional battery cell with a lithium reference electrode for 50 cycles. The same cross sections of the spherical particles were compared before and after the cycles, as shown in Figure 4a–h. The corresponding voltage profiles for both NCM materials are plotted in Figure S5 in the Supporting Information. After 50 cycles, the electrodes were washed gently, and the cross sections of the spherical particles were cut slightly again by FIB to remove the residual electrolyte from the exposed surface. This process explains the absence of some of the cracks previously observed in the pristine state (Figure 4a,b) in the image taken after the cycles (Figure 4c,d).

**Figure 4:**
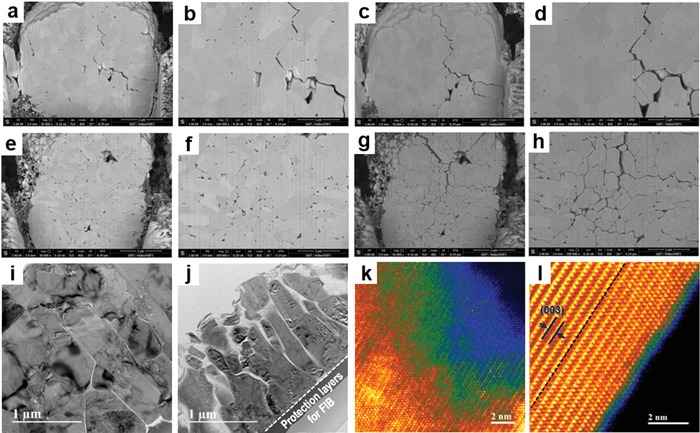
Ex situ SEM and TEM analyses of the structural changes of NCM cathodes. SEM cross‐sectional images of traced secondary particles of a–d) NCM 111 and e–h) NCM 811 cathode materials acquired a,b,e,f) before and c,d,g,h) after 50 electrochemical cycles of the half‐cell test. Low‐magnification TEM images of i) NCM 111 and j) NCM 811 cathode materials after 300 electrochemical cycles of the NCM/graphite full cell test. k,l) Atomic‐resolution HAADF‐STEM images obtained near the surface of the 300‐cycled primary‐like particles of the NCM 811 cathode. The images are colored according to the temperature color table provided by the Gatan DigitalMicrograph software; the colors range from black, through blue, green, red, and yellow to white. l) Cation intermixed layers near the surface are separated by black dashed lines.

The cross‐sectional SEM images of the pristine materials (Figure 4a,b,e,f) clearly show the difference in packing density in addition to the size difference of the primary‐like particles between the two NCM materials, both of which may be attributed to the relative intrinsic instability of the Ni‐rich NCM materials.^4^, ^5^, ^6^, ^22^ The structural deterioration of the two cathodes after 50 cycles was also very different (Figure 4c,d,g,h). Microcracks developed between the primary‐like particles of both materials. Most of the primary‐like particles remained well integrated in NCM 111 (Figure 4c,d), but considerable cracks developed in NCM 811, and crevices were noticeable in most of the primary‐like particle surfaces (Figure 4g,h). The microcrack propagation is generally ascribed to the repetitive and significant volume expansion/contraction of the materials during the cycles, ultimately causing the structural and performance degradation of Ni‐rich layered cathodes.^8^, ^20^, ^35^, ^36^, ^37^, ^38^, ^39^ We further compared the TEM images of electrochemically cycled NCM 111 and NCM 811. Figure 4i–l shows the cross sections of the primary‐like particles in the micrometer‐sized spherical particles of NCM 111 and NCM 811 that were electrochemically charged and discharged for 300 cycles at 1 C current rate in NCM/graphite full cell batteries. The TEM samples were made using the FIB sampling technique and fine thinning at a low voltage to avoid unexpected damage. The large crevices seen in the cycled NCM 811 cathode (Figure 4j) are the microcracks developed between the primary‐like particles, which are also seen in the ex situ SEM experimental results in Figure 4g,h. On the contrary, the NCM 111 cathode maintained its original morphology very well (Figure 4i), as also shown in Figure 4c,d.

Additionally, the atomic‐resolution HAADF‐STEM image of the primary‐like particle surface of the NCM 811 cathode in Figure 4k indicates severe surface dissolution and deterioration after the cycles, which may also increase the performance degradation of the Ni‐rich NCM cathode. The transition‐metal ion migration into the lithium layers, which transforms the α‐NaFeO_2_ structure into a metal oxide rock salt structure, was extensive over a wide region near the surface. In addition, the different colors in the temperature color‐coded HAADF‐STEM image, which indicate variations in thickness, imply that the significant elemental dissolution from the surface formed several terrace layers. In contrast, structural collapse was not severe on the surface facing the MO_2_ slabs even in the Ni‐rich NCM, as shown in Figure 4l. The surface along this direction is as flat as the pristine material, and transition‐metal ion migration into the lithium layers is limited to the surface. This behavior may be caused by the much lower surface energy of the {003} slab planes, compared to the planes where the lithium‐ion diffusion channels are exposed (the {10−2} or {*hk*0} planes) in the Ni‐rich NCM cathode^40^; accordingly, the latter planes break easily during the cycles. This is consistent with the fact that most primary‐like particles of the NCM 811 material have a long finger‐like shape, as shown in Figure 2e–h. We confirmed that the {003} planes are mostly exposed on the wide flat surface of the finger‐shaped primary‐like particles of NCM 811, which demonstrates the serious imbalance in the surface energies of the two types of surface planes.

Most importantly, we demonstrated that nanocracks develop even in a single primary‐like particle of Ni‐rich layered cathodes after prolonged cycles, which highlights the necessity of redefining primary particles. **Figure** **5** shows a representative example of the nanocrack that developed in a single primary‐like particle of NCM 811. As shown in Figure 5a, the particles separated upward and downward share a very similar outer border shape, which could originate from a single primary‐like particle that is forced to crack vertically. The well‐fitted boundary line between the two separated particles strongly supports the formation of a crack inside the single primary‐like particle during the electrochemical cycles. We performed HRTEM‐FFT analysis of the nearby area to reveal where the cracks occur in the primary‐like particle. Figure 5b,c,e,f shows the FFT patterns derived from the HRTEM images (Figure S6, Supporting Information) of the zone axis aligned with the upper fragment. The FFT patterns of the upper particle (Figure 5b,c) are well indexed with the [120] plane of the conventional α‐NaFeO_2_‐structured NCM material. The core of the particle (Figure 5b) has almost the same crystal structure as the pristine state with no transition‐metal ion migration into the lithium layer. However, the (003) peaks disappeared near the crack point (Figure 5c), which indicates that the layered α‐NaFeO_2_ structure was transformed into the rock salt structure owing to severe transition‐metal ion migration, similarly to the primary‐like particle surfaces. The atomic‐resolution HAADF‐STEM images shown in the insets of Figure 5b,c confirm the atomic structures, and the corresponding atomic model structure is presented in Figure 5d. However, it was found that the lower particle has a slightly different orientation of [11 22 1] at the same projection, as shown in Figure 5e,f. Diffraction spots from the (2−10) planes were only observed near the crack point possibly because of the partial collapse of the structure. The arraying direction of the lower particle was confirmed by the SAED pattern following after a small tilt to realign the lower particles along the lower‐index zone axis (Figure S7, Supporting Information). The SAED pattern taken after a 5° rotation according to the left‐hand rule (left thumb pointing in the virtual vertical direction) in Figure S7c in the Supporting Information was indexed as the NCM [8 16 1] zone axis. The atomic‐resolution HAADF‐STEM image obtained at this zone axis (Figure S7b, Supporting Information) agrees well with the atomic model structure of this projection direction. The atomic model structures of the upper and the lower particles (Figure 5d,g) illustrate the original relationship between the particles before the crack development; the lithium‐ion diffusion channels border each other with slight tilting angles.

**Figure 5:**
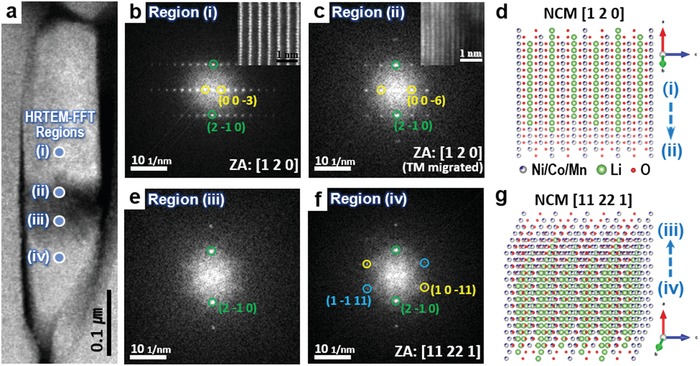
HRTEM analysis of nanocrack points identifying the region where the crack occurs in a primary‐like particle of Ni‐rich NCM cathodes. a) HAADF‐STEM image of a nanocrack developed in a primary‐like particle after 300 cycles in an NCM/graphite full cell battery. b,c,e,f) FFT patterns from the HRTEM images (Figure S6, Supporting Information) of regions (i) to (iv). b,c) Corresponding atomic‐resolution HAADF‐STEM images are shown in the insets. Atomic model structures of d) regions (i) and (ii) and g) regions (iii) and (iv).

Nanocracks can develop at the GBs between the true primary particles within a single primary‐like particle after severe deterioration of the material, and this cracking can finally break the primary‐like particle into two smaller particles. Our observation reveals, for the first time, nanocracks can evolve in a so‐called primary particle of Ni‐rich layered cathodes during prolonged cycles between the typical operating voltage ranges. The formation mechanism of the nanocracks may be similar to that of the microcracks between the primary‐like particles; the repetitive volume expansion/contraction of two proximal primary particles with slightly different orientations may result in the accumulation of defects and strains at the GB during the cycles, which causes a strain‐induced nanocrack in the primary‐like particle. Indeed, we frequently observed transition‐metal ion migration at the GBs in the primary‐like particles after the prolonged cycles (see Figure S8 in the Supporting Information for a representative example of structural transformation near the GBs), as on the surface of primary‐like particles. This migration explains the higher probability of nanocrack development in the relatively unstable Ni‐rich layered cathodes. We also observed a substantial decrease in the size of the primary‐like particles—to almost half their original size—around significantly degraded parts of the NCM 811 cathode after 300 cycles in the full cell battery (Figure S9, Supporting Information). This decrease demonstrates the vigorous nanocrack development in the Ni‐rich NCM cathode, which breaks the primary‐like particles into smaller ones. The nanocracks inside the primary‐like particles should also be considered as an important factor contributing to the degradation of the Ni‐rich layered cathodes and causing the loss and isolation of primary particles. The low critical energy release rates for crack generation in Ni‐rich NCM materials calculated by density functional theory^39^ also support its fragile characteristic. There has been a report that found intragranular cracks in an NCM cathode developing at high‐voltage cycling,^41^ which originate from the amplified local strain between specific MO_2_ slabs induced by inhomogeneous and deep Li‐ion extraction during high‐voltage charging. The intragranular cracks are also strain‐induced, but clearly distinctive from the nanocracks that are caused by repetitive anisotropic volume expansion and contraction of two proximal misoriented grains within primary‐like particles that develop in the typical operating voltage range. The progress of microcrack and nanocrack development in the Ni‐rich NCM cathode is briefly illustrated in **Figure** **6**. In addition to providing a basis for the severe structural degradation of Ni‐rich layered cathodes, the observation of nanocracks helped elucidate the structure of the micrometer‐ and nanometer‐sized particles of layered lithium transition‐metal oxide materials.

**Figure 6:**
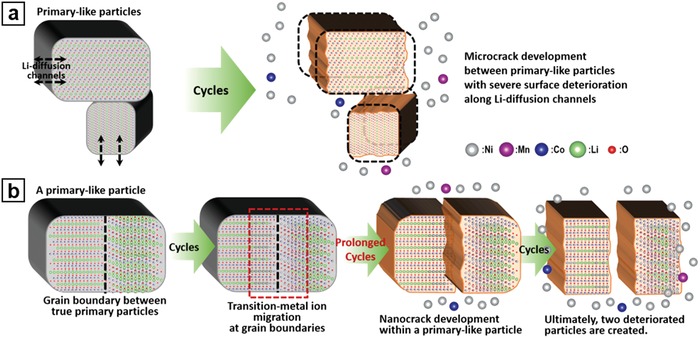
Schematic illustration of crack development in Ni‐rich NCM cathode materials. a) Microcrack development between primary‐like particles and b) nanocrack development within a single primary‐like particle.

In summary, we examined the so‐called primary particles of NCM materials, which are representative layered lithium transition‐metal oxides for lithium‐ion battery cathodes, by elaborate electron microscopy studies. Particles with sizes ranging from hundreds of nanometers to 1 µm packed in a ≈10 µm spherical particle of layered lithium transition‐metal oxides have been regarded so far as single‐crystal primary particles. However, scanning precession NBD and atomic‐resolution STEM analyses revealed that the so‐called primary particles are in fact polycrystalline secondary particles. The secondary particles are formed by the aggregation of smaller true primary particles by oriented attachment and contain low‐angle and exceptionally stable coherent symmetrical (special) GBs that allow a lattice mismatch to some extent. We renamed this so‐called primary (secondary, in fact) particle as “primary‐like particle.” Nanocracks can develop at the GBs between the true primary particles within a primary‐like particle after repetitive electrochemical cycles, which contribute to the structural and performance degradation of the Ni‐rich layered cathodes besides the microcracks developed between the primary‐like particles. In addition to providing a thorough understanding of the micro‐ to atomic structure of layered lithium transition‐metal oxide materials, this study suggests that Ni‐rich layered cathodes with superior performance can be achieved by controlling the true primary particles. For example, reducing the number of GBs in primary‐like particles or surface treatment of the true primary particle may limit structural degradation from the mechanical cracks. We expect that this study will contribute to the development of Ni‐rich NCM cathode materials.

## Experimental Section

*Sample and Coin Cell Preparation and Electrochemical Measurement*: The NCM 111 and NCM 811 materials were prepared by a coprecipitation method followed by solid‐state sintering. Spherical particles of the transition‐metal hydroxide precursors (Ni_0.33_Co_0.33_Mn_0.33_)(OH)_2_ and (Ni_0.8_Co_0.1_Mn_0.1_)(OH)_2_ were synthesized by the coprecipitation method, and a mixture of the precursor and LiOH·H_2_O powders was sintered to obtain NCM 111 and NCM 811 powders. The details of the procedure are described elsewhere.^35^ NCM 111 and NCM 811 were used as cathodes for the NCM/graphite full cell test. Graphite and a porous polyethylene‐based membrane were used as anode and separator, respectively. 1.3 mol L^−1^ LiPF_6_ dissolved in ethylene carbonate and dimethyl carbonate (1:1 by weight) was used as the electrolyte solution. The CR2032 coin cells were assembled in a dry room. Electrochemical tests were performed using a battery cycler (VSP‐300; Bio‐Logics). The cells were charged/discharged at 1 C current rate in the voltage range of 2.8–4.35 V.

*Transmission Electron Microscopy*: Cross‐sectional TEM specimens were prepared from ≈10 µm spherical particles of the pristine and the cycled electrodes using the FIB sampling technique. Crystallographic orientation maps were obtained with a JEOL JEM‐2100F 200 kV field‐emission TEM instrument equipped with an ASTAR (NanoMEGAS) device. All other TEM data (high‐resolution TEM/STEM images, electron diffraction patterns) were acquired with a double aberration‐corrected JEOL ARM‐200CF TEM instrument with a cold‐field emission gun operated at an acceleration voltage of 200 kV.

*Ex Situ Scanning Electron Microscopy*: To trace a single particle before and after electrochemical cycles, a reference location was marked on the NCM cathode using a pulse laser prior to cell cycling. Cross sections of selected pristine secondary particles were produced by FIB and observed by SEM. After the SEM observation, CR2032 coin‐type half‐cells were assembled with the NCM particles, separator, lithium metal, and electrolyte. The cell was disassembled, washed, and dried in a dry room after 50 cycles under 0.5 C current rate. Then, the microstructural evolution of the NCM particles was observed, as shown in Figure 4a–h.

## Conflict of Interest

The authors declare no conflict of interest.

## Supporting information

Supplementary
