# Supplementary material for: Revisiting Primary Particles in Layered Lithium Transition‐Metal Oxides and Their Impact on Structural Degradation
Source: Adv Sci (Weinh). 2019 Jan 25;6(6):1800843. doi: 10.1002/advs.201800843 (PMC6425450; doi:10.1002/advs.201800843)
Supplement: Supplementary file 1 — Supplementary [file ADVS-6-1800843-s001.pdf]

Copyright WILEY-VCH Verlag GmbH & Co. KGaA, 69469 Weinheim, Germany, 2019.

Supporting Information

for *Adv. Sci.,* DOI: 10.1002/advs.201800843

Revisiting Primary Particles in Layered Lithium Transition-Metal Oxides and Their Impact on Structural Degradation

Seung-Yong Lee, Gyeong-Su Park, Changhoon Jung, Dong-Su Ko, Seong-Yong Park, Hee Goo Kim, Seong-Hyeon Hong, Yimei Zhu,* and Miyoung Kim**

Copyright WILEY-VCH Verlag GmbH & Co. KGaA, 69469 Weinheim, Germany, 2019.

Supporting Information

Revisiting Primary Particles in Layered Lithium Transition-Metal Oxides and Their Impact on Structural Degradation

Seung-Yong Lee, Gyeong-Su Park*, Changhoon Jung, Dong-Su Ko, Seong-Yong Park, Hee Goo Kim, Seong-Hyeon Hong, Yimei Zhu*, and Miyoung Kim*

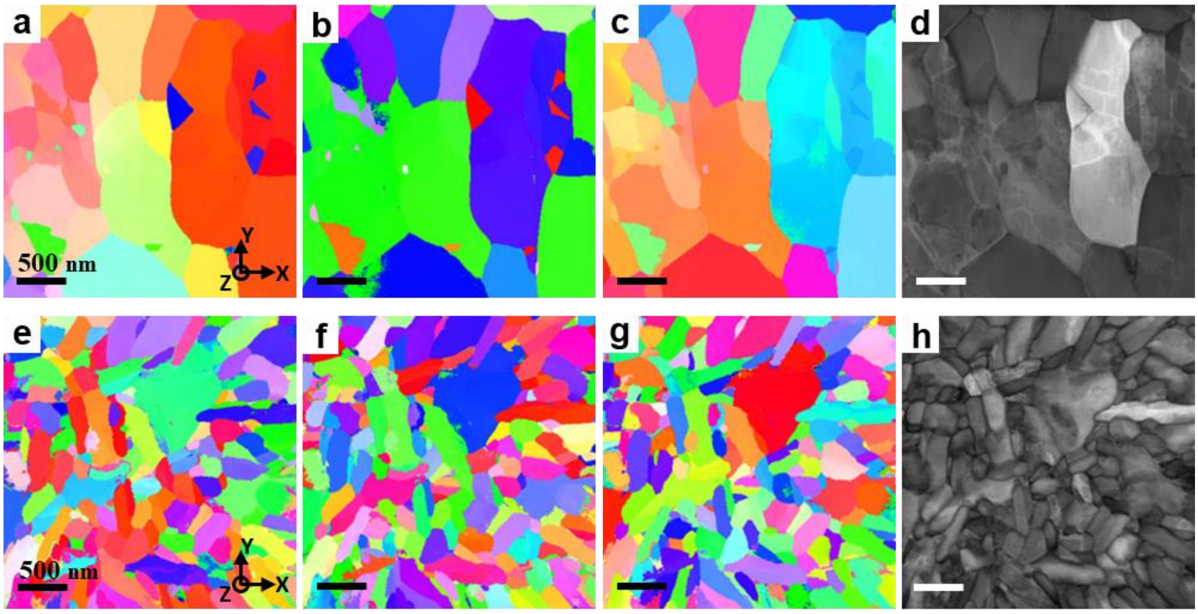

Figure S1. (a–c, e–g) Raw crystal orientation maps obtained from pristine (a–c) NCM 111 and (e–g) NCM 811, corresponding to Figure 2b–d and 2f–h, respectively. (d, h) Index maps of (d) NCM 111 and (h) NCM 811 combined with the raw crystal orientation maps in Figure 2b–d and 2f–h. The index map indicates the matching index between the experimentally obtained NBD pattern and the corresponding theoretical diffraction pattern from the given crystal structural model. Brighter positions have higher matching indices.

As shown in Figure S2, all of six particles have slightly different precession NBD patterns between their starting and the end points. They show distinct features of small-angle misorientations. For examples, precession NBD patterns from Particle 3 in Figure S2g and S2h have similar diffraction spots attributed to the additional spots from high-order Laue zone (HOLZ), but their intensities are different due to the slight misorientation of the projecting directions, close to [-8-7-2] and [-9-7-2],

respectively. Influence of dynamical electron diffraction on the diffraction spot intensities can be excluded due to the electron beam precession effect, which makes it easier to specify the precise crystal orientations. In addition, it is obvious that Particles 1 and 5 have slight in-plane misorientations between the starting and the end points at the same [-1-10] projecting direction. It can be better recognized by the merged images shown in Figure S2c and S2o.

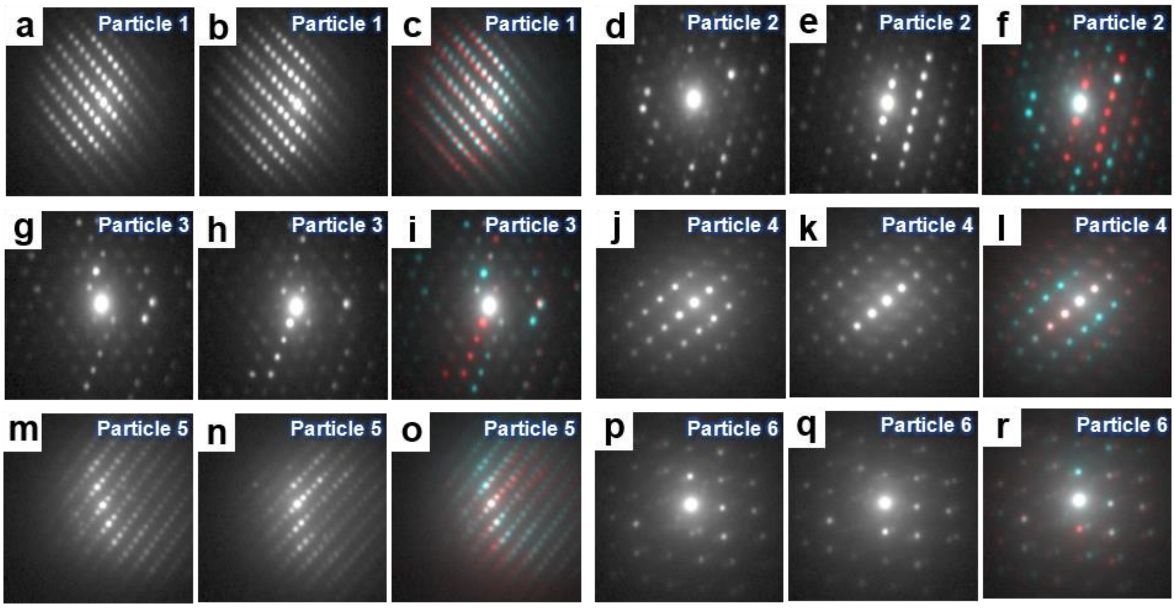

Figure S2. Precession nanobeam electron diffraction (NBD) patterns collected for crystallographic orientation mapping of pristine NCM 111 and NCM 811 materials in Figure 2, acquired using ASTAR device. Raw precession NBD patterns at (a, d, g, j, m, p) the starting points (dots) and (b, e, h, k, n, q) the end points (arrowhead) of yellow dashed arrows for each particle marked in Figure 2d and 2h are shown as representatives. (c, f, i, l, o, r) Merged images of NBD patterns at the starting and the end points of each particle to compare the two patterns. NBD patterns at the starting and the end points are indicated with sky-blue and red colors.

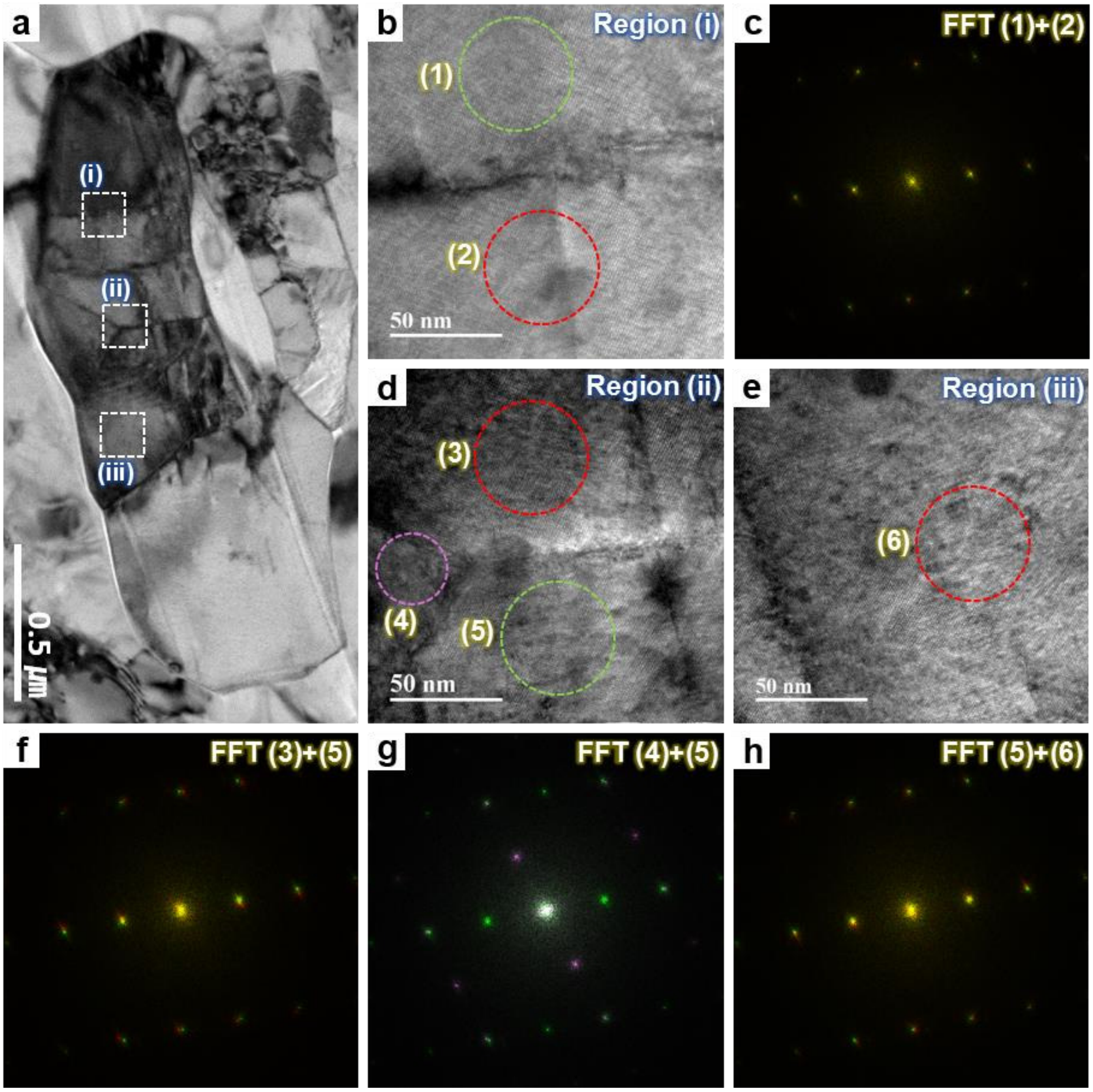

Figure S3. High-resolution TEM (HRTEM) images of a primary-like NCM 111 particle, corresponding to the particle shown in Figure 3. (a) A low-magnification TEM image of the primary-like particle. (b, d, e) HRTEM images at Regions (i), (ii), and (iii) indicated in (a). The Regions (i), (ii), and (iii) are identical to the Regions marked in Figure 3 with same symbols. (c, f, g, h) Merged FFT patterns from two different locations nearby. FFT patterns obtained locations are indicated by dashed circles with corresponding colors and Arabic numbers in round brackets in (b), (d), and (e). (c, f, h) Low-angle in-plane misorientations between the neighboring grains show the prevalent low-angle grain boundaries in a so-called primary particle. (g) Special in-plane orientation relationship between the two nearby grains shows the origin of coherent symmetrical boundaries.

Figure S4 shows another primary-like particle containing coherent symmetrical boundaries. The particle shown in the low-magnification TEM image of Figure S4a has the common shape of a so-called primary particle. The GB lines are clearly observed in the low-magnification TEM image (Figure S4a) and high-resolution HAADF-STEM image (Figure S4b), which are spread along the directions similar to the coherent symmetrical boundaries shown in Figure 3a and 3e. Atomic-resolution HAADF-STEM images in Figure S4c and S3d confirm the atomic structures of these GBs, i.e. coherent symmetrical boundaries, identical to the special GBs shown in Figure 3e. These distinct GBs are the clear evidence demonstrating the prevalence of coherent symmetrical GBs and sub-grains in so-called primary particles, which clarify the misconception on the so-called primary particles so far.

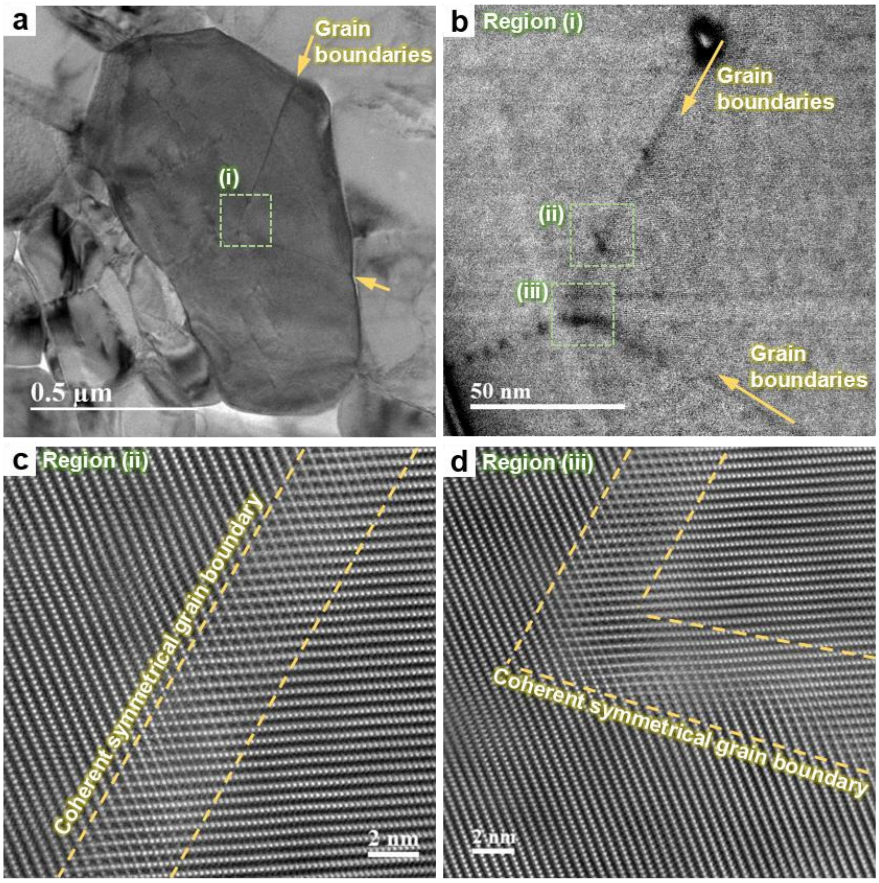

Figure S4. Another example of coherent symmetrical special grain boundaries (GBs) observed in a so-called primary NCM 111 particle. (a) A low-magnification TEM image of a so-called primary NCM particle. (b) A high-resolution HAADF-STEM image at Region (i) indicated in (a). Dark lines in

the image are grain boundaries, the orientation relationship of which is similar to the special GBs shown in Figure 3e. (c, d) Atomic-resolution HAADF-STEM images at Regions (ii) and (iii) indicated in (b). Coherent symmetrical special GBs having an $MgAl_2O_4$-type spinel structure are formed between the nearby grains, which are similar to the example shown in Figure 3e. This figure shows that the special GBs can be frequently developed in so-called primary NCM particles, which clearly demonstrates the prevalent sub-grains in the so-called primary particles.

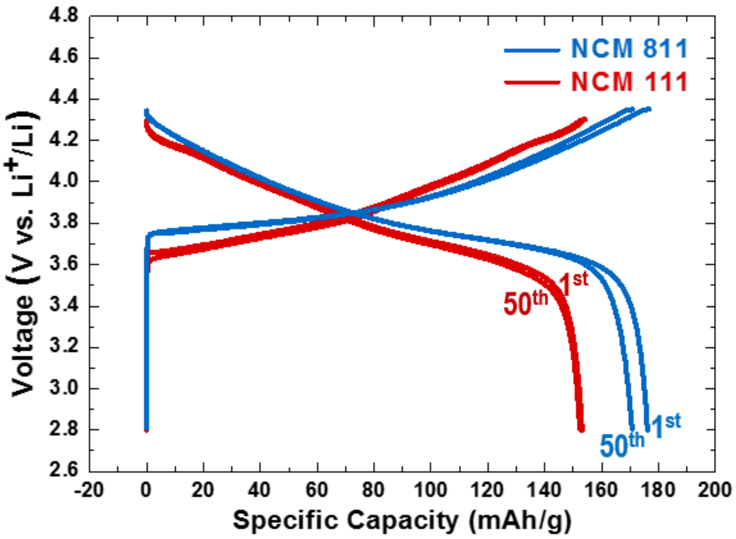

Figure S5. Charge/discharge profiles of the 1st and 50th cycles of NCM 111 and NCM 811 cathodes obtained for the ex situ SEM experiment, which correspond to Figure 4a–h. The NCM cathodes were electrochemically cycled in a half-cell with a lithium metal anode at a 0.5C current rate. During the charge, the cutoff voltage was retained until the current rate reached 0.1C.

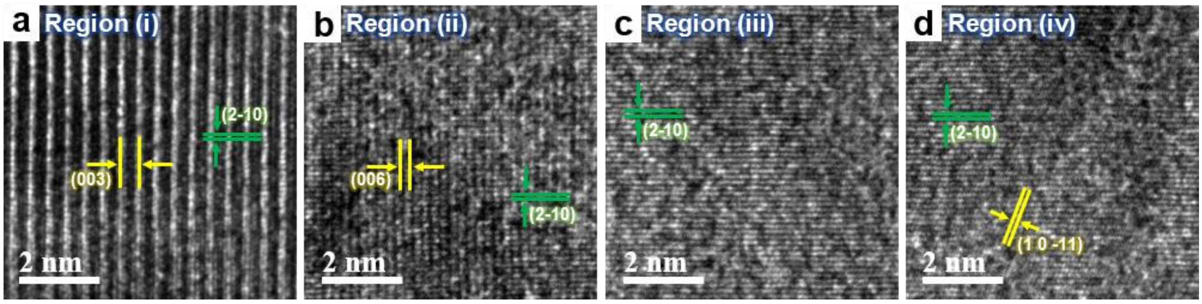

Figure S6. HRTEM images corresponding to the regions indicated in Figure 5a and FFT patterns shown in (a) Figure 5b, (b) Figure 5c, (c) Figure 5e, and (d) Figure 5f.

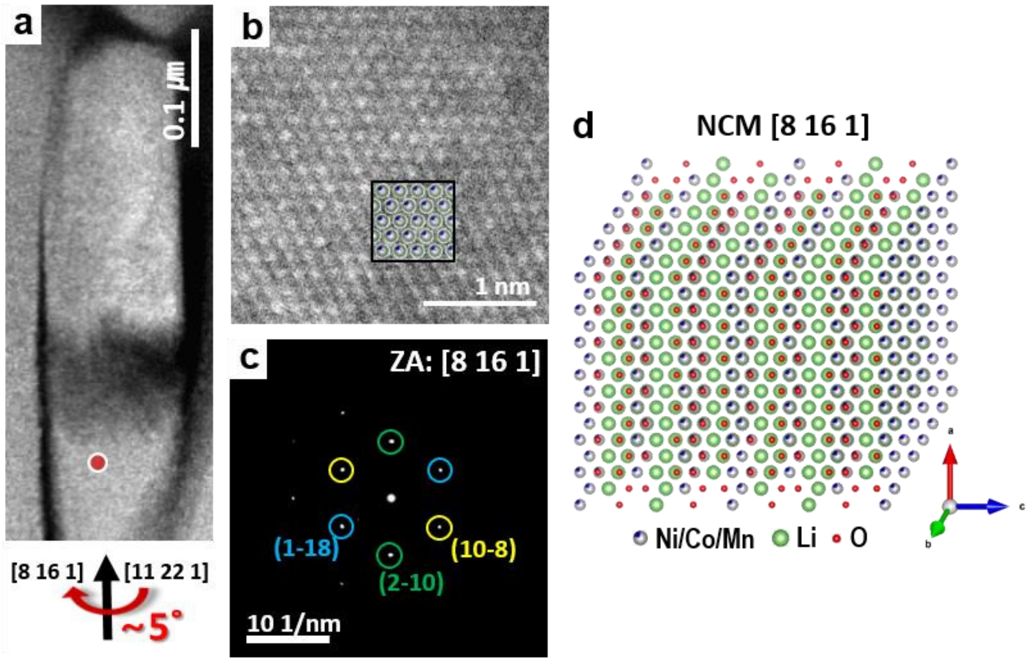

Figure S7. Five-degree tilted view from Figure 5e–g. (a) HAADF-STEM image shown in Figure 5a. (b) HAADF-STEM image and (c) selected area electron diffraction pattern acquired at the region corresponding to Figure 5f after a 5° tilt according to the left-hand rule (the left thumb pointing in the virtual vertical direction). The location where the STEM image and diffraction pattern were acquired is marked with a red dot in (a). The tilting orientation from Figure 5 is also indicated at the bottom of (a). The corresponding atomic model structure is superposed in (b). (d) Atomic model structure corresponding to (b) and (c). Lithium, oxygen, and transition-metal ions cannot be distinguished at this projection direction because of the overlaps.

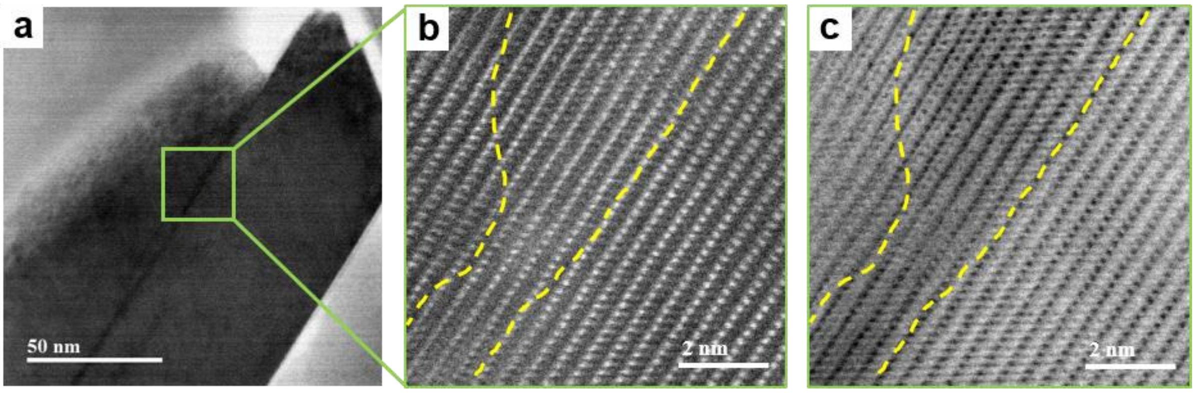

Figure S8. Noticeable transition-metal ion migration at a low-angle GB after electrochemical cycles. (a) Low-magnification ABF-STEM image of the GB region. (b) HAADF-STEM and (c) ABF-STEM images showing the remarkable transition-metal ion migration into the lithium layers near the GB.

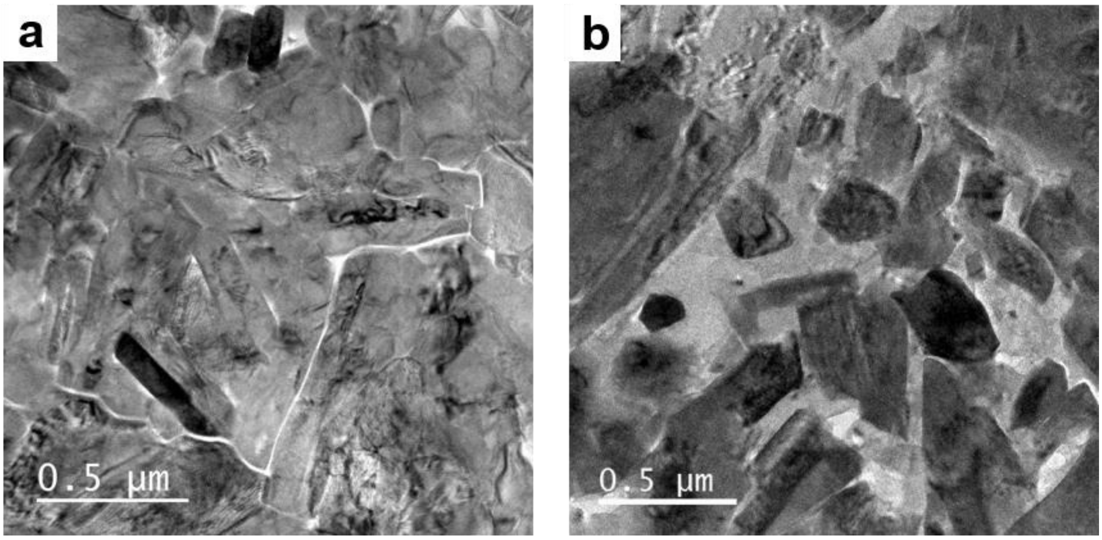

Figure S9. TEM images of a (a) pristine and (b) severely degraded region of the 300-cycled NCM 811 cathode in a NCM/graphite full cell battery. The TEM samples were prepared by the FIB sampling technique.
